# Gerontechnology for better elderly care and life quality: a systematic literature review

**DOI:** 10.1007/s10433-023-00776-9

**Published:** 2023-06-22

**Authors:** Genghua Huang, Samuel Ampadu Oteng

**Affiliations:** 1 School of Graduate Studies and Institute of Policy Studies, Lingnan University, 8 Castle Peak Road, Tuen Mun, New Territories, Hong Kong; 2 School of Graduate Studies, Lingnan University, 8 Castle Peak Road, Tuen Mun, New Territories Hong Kong

**Keywords:** Gerontechnology, Older persons, Social caregivers, Health-related well-being, Life quality

## Abstract

Gerontechnology as multidisciplinary research has expanded in recent years due to its significant role in ensuring better care and improved quality of life for older adults and their caregivers. With a substantial increase in studies on reasons behind less inclination of older individuals to accept gerontechnology, barriers to its non-acceptance appear to be persistent. In addition, there is a dearth of research on the adoption of gerontechnology from the perspectives of social caregivers, given that caregivers bear a substantial burden in the form of chronic stress, which adversely affects their health and that of older people. Therefore, the aim of this study is to present a holistic perspective of older adults and their caregivers by systematically reviewing literature on gerontechnology acceptance. Adopting the preferred reported items for systematic and meta-analysis (PRISMA) framework, publications specifically on gerontechnology from 2002 to 2022 in Scopus, Web of Science and PubMed, that focused on older people (50 years and above) and caregivers (informal and formal) were reviewed. We critically evaluated 25 publications and synthesised them thematically. The results highlight that gerontechnology acceptance by older adults and their social caregivers is highly contingent on certain personal, physical, socio-cultural and technological indicators. However, this paper concludes that a generalised policy approach for gerontechnology and a better quality of life may be ineffective, considering that older adults and social caregivers constitute two heterogeneous groups.

## Introduction

One of the greatest global concerns for healthcare and social institutions is the ageing population (WHO 2018). While demographic trends differ among countries and regions of the world, the ageing population is increasingly becoming a challenge in both developed and developing countries (Scott et al. 2019). Policymakers have proposed gerontechnology: a creative, multidisciplinary solution to deal with this challenge by linking ageing and technology. Conceptually, gerontechnology denotes a scientific study of ageing, examining the biological, psychological and sociological factors associated with the ageing process (Halicka and Surel 2021), which can help older adults identify and slow down the effects of age-related physical and cognitive difficulties (Sale 2018). Gerontechnology therefore has enormous potential to ensure better care and improved quality of life (QoL) for older adults.

Although gerontechnology is supportive of daily life, it is widely recognised that older people do not show as much interest in adopting new technologies as younger populations (e.g. Gullà et al. 2015; Wu et al. 2015; Yusif et al. 2016). Several studies have been conducted over the last few decades to investigate the numerous reasons why older individuals are less inclined to use gerontechnology (e.g. Berkowsky et al. 2017; Chen and Chan 2014). However, barriers to the non-acceptance of gerontechnology by older people appear to be persistent (Lee and Tak 2022). This is attributable to a misconception as to which gerontechnologies are desirable for older adults, as well as the factors and perspectives that determine their usage or non-usage (Harris et al. 2022). In addition, social caregivers often play a vital role in assisting community-dwelling older adults in managing their health (Bevilacqua et al. 2020; Papetti et al. 2014). Studies have demonstrated that the growing, caring needs of an ageing society can be met by increasing not only the technologies developed to assist older people but also the number of carers (Cook et al. 2020; Robinson et al. 2020). By virtue of this, past studies have established that gerontechnology can be a useful tool for caregivers in several ways, including peer networking, professional support and resource identification (Hopwood et al. 2018; McHugh and Lawlor 2012). Furthermore, caregivers bear a substantial burden in the form of chronic stress, which adversely affects their health and that of older people (Adelman et al. 2014). However, there is a dearth of research on the adoption of gerontechnology from the perspectives of social caregivers. Most previous studies exploring caregivers’ perspectives have focused on telehealth and assistive technologies for all populations (e.g. Cook et al. 2018; Mostaghel 2016; Peek et al. 2014).

Given the important role of social caregiving, the perceived needs and challenges of gerontechnology in older adults and their social caregivers remain important for social policy interventions. Until now, no systematic review of gerontechnology has provided evidence for these two different groups. In our study, instead of focusing only on older adults, the main objective is to systematically review evidence on the opinions of older adults and social caregivers on gerontechnology acceptance. Secondly, this review also discusses the effects of gerontechnology on better QoL and social caregiving for older adults. We believe our findings will benefit various stakeholders, such as designers, engineers and researchers, to study and fully develop gerontechnology products and services. This, in turn, will reduce the care burdens of social caregivers and enhance the QoL for older adults, particularly in regions where the ageing population is rising exponentially.

## Methods

This systematic review was carried out in accordance with the suggested step-by-step strategy outlined in the PRISMA guidelines (Moher et al. 2009), ensuring the reliability, usefulness and scientific soundness of the review (Hale and Griffiths 2015). A detailed description of the procedure is provided in the following subsections.

### Search strategy

We conducted an extensive search in Scopus, Web of Science and PubMed to cover empirical studies that reported on the adoption of gerontechnology among older people (50 years and above) and caregivers (informal caregivers and nursing homes). These databases were selected due to their prominence and contributions to ageing, gerontechnology and geriatrics issues. The review specifically focused on four broad search terms: (1) ‘gerontechnology’, (2) ‘adoption’, (3) ‘older adults’ and (4) caregivers. While some reviews (e.g. Mostaghel 2016; Peek et al. 2014; Yusif et al. 2016) had focused on the generic term ‘technology’, we specifically focused on the terminology ‘gerontechnology’ to ensure that those publications captured are specifically focused on the technologies to assist older adults. To keep our search as broad as possible, the review limited the search string to the fields of title, abstract and keywords in each database. The combination of the key terms with Boolean operators, for example, in Scopus, included: (‘gerontechnology’) AND (‘adoption’), (‘gerontechnology’) AND (‘caregivers’) OR (‘social care’), (‘gerontechnology’) AND (‘health’), (‘gerontechnology’) AND (‘well-being’) OR (‘quality of life’) OR (‘happiness’) OR (‘life satisfaction’). Table 1 explains how the final search string of the key terms used in the review was arrived at. To ensure a comprehensive search, the review was limited to a 20-year publication period (2002–2022) in all databases.

**Table 1:** Search string of key terms

Order of search	Terms
1	Gerontechnology AND adoption
2	Gerontechnology AND caregivers OR social care
3	Gerontechnology AND health
4	Gerontechnology AND quality of life OR wellbeing OR happiness

### Eligibility criteria

Guided by the aims and objectives of the review, all publications were subjected to predetermined inclusion and exclusion criteria. Studies were included if they examined the reasons for the adoption of gerontechnology. Studies that examined whether and how gerontechnology reduces caregivers’ burden were also included. In addition, studies that were empirical, employing qualitative, quantitative or both methodologies, and written or published in English were also included. Furthermore, those studies that considered older people or/and caregivers as study populations were included. Following the inclusion criteria, the review excluded studies that were focused on technologies for all populations and not on gerontechnology as an intervention for reducing caregivers’ burdens. Moreover, empirical studies that involved individuals or groups other than older people and caregivers were excluded. The review also excluded reviewed papers, theoretical and conceptual articles. Lastly, articles published in a non-English language were in the exclusion criteria.

### Screening

In the initial stage, the database search was conducted by two independent researchers (HG and OSA). Subsequently, the studies’ titles and abstracts were screened based on the predetermined inclusion and exclusion criteria. Then, an expert researcher was consulted to resolve all discrepancies in the required studies that met the inclusion criteria. All outstanding issues were discussed with the two researchers and resolved under the supervision of the expert researcher. All studies were exported into Microsoft Excel software, where duplicates were eliminated. In the final stage, the full text of the various studies was screened after duplication removal. Two independent researchers managed the screening at this stage and decided which studies should finally be included in the review.

### Data extraction and quality appraisal

After the final screening stage, a data extraction guide on the topic was developed following previous studies (e.g. Merkel and Kucharski 2019; Sundgren et al. 2020). The two independent researchers developed separate extraction templates for the articles; however, upon discussion, consensus was reached, and the templates were aggregated into one data extraction template (see Table 2). The methodological validity of all publications selected for inclusion were evaluated using the Mixed Methods Appraisal Tool, 2018 version (MMAT). The appraisal tool was selected because it was designed for the appraisal of systematic reviews that include qualitative, quantitative and mixed methods studies (Hong et al. 2018). Besides, it is extensively clear, designed to allow the authors evaluate the reliability and validity of all included publications. Therefore, for each study, a scoring logic of ‘yes’ was assigned as an indication of satisfying a quality criterion. Any study that received a “no” in the scoring logic did not meet the quality standards. On the other hand, if the paper did not present sufficient information to determine whether a criterion was met, or if the information reported was ambiguous, then the study was assigned the “Can't tell” response (see Table 3). The authors followed up for supplementary papers or contacted the authors to request for more clarification. As prescribed in the MMAT guidelines (Hong et al. 2018), a sensitive analysis of contrasting the results of the ratings of each criterion was followed to better inform the quality of all included studies.

**Table 2:** Catalogue of included studies

No.	References	Study Setting	Research objective	Methods	Theoretical model	Main results
1	Huang et al. (2021)	China	To explore the intention of Chinese community-dwelling older adults to adopt gerontechnology and its influencing factors	Mixed-methods approach; sequential explanatory design Phase 1: Questionnaire—multifactor logistic regression *N* = 1180 Phase 2: Semi-structured interview—thematic analysis *N* = 18		Most older adults showed adoption intention towards gerontechnology. Predicting, enabling and need factors influenced adoption of gerontechnology.
2	Joseph et al. (2018)	Malaysia	To examine the factors of older adults’ adoption intention of gerontechnology (mobile bathtubs)	Mixed-methods approach Qualitative: focus group discussion *N* = 12 Quantitative: survey *N* = 37 Age: not reported		The findings show that perceived ease of use and perceived usefulness, along with specific design features of mobile bathtub were determinants of adoption intention among older adults.
3	Chen and Chan (2014)	Hong Kong	The aim of this study was to examine the factors that influence the acceptance of gerontechnology by older Hong Kong Chinese	Quantitative: survey *N* = 1012 (seniors) Age = 55+ years	Technology acceptance model (TAM) unified theory of acceptance and use of technology (UTAUT)	The models adopted in this study proved useful. However, in contrast to TAM and UTAUT, significant effects for perceived usefulness, perceived ease of use and attitude towards using the technology on usage behaviour were not found in this study. Personal attributes like technology self-efficacy, anxiety and facilitating conditions (FCs) were more decisive than perceived benefits for predicting gerontechnology usage behaviour.
4	Chen and Chan (2013)	Hong Kong	To explore the attitudes and experiences of older people towards using gerontechnology and determine the factors accounting for its use and non-use	Qualitative: Interviews: *n* = 26 Focus group discussion: *n* = 24 *N* = 50 Age = 55–85 years		Positive attitudes were most frequently related to enhanced convenience and advanced features. Negative attitudes were most frequently associated with health risks and social problems arising from using technology. Outcome expectations, social influences (SIs) and support from facilitators influenced usage, whereas non-use of gerontechnology relates to the personal, technological and environmental factors that lead to non-usage.
5	Halicka and Surel (2021)	Poland	To determine the most desired group of gerontechnologies among current and trend users	Quantitative: survey *N* = 1152 Age = 40+		Most desirable gerontechnology was health related and selected primarily based on its innovativeness and not as result of its usage.
6	Delbreil and Zvobgo (2013)	Switzerland and France	The purpose of the study was to examine health professionals’ recognition of sensor technology as a means to enhance quality of life (QoL) of care recipients with dementia	Mixed-methods approach: interviews and questionnaires Multiple regression analysis *N* = not reported	Technology acceptance model (TAM)	Positive attitude towards gerontechnology as a means to enhance QoL of older persons.
7	Khan et al. (2021)	Pakistan	To investigate the elderly’s intention to adopt mobile phone technology for healthcare (mHealth)	Quantitative: survey *N* = 286 Structural equation modelling	Unified theory of acceptance and use of technology (UTAUT)	Performance expectancy (PE), effort expectancy (EE), social influence (SI), facilitating conditions (FCs), perceived ubiquity (PU), and perceived trust (PT) have a positive significant relationship with mHealth adoption intention (AI). The results do not indicate a negative relationship between technological anxiety (TA) and mHealth AI. Gender significantly moderates the relationship between PE and SI and mHealth AI.
8	Chen et al. (2021)	USA	To understand barriers and design opportunities to improve healthcare and QoL for older adults through voice assistants	Qualitative: interviews: *N* = 21 *n* = 16 (older persons) *n* = 5 (caregivers)		The study highlights challenges in the designing of intelligent voice assistants (IVAs) for older adults, especially for healthcare-related tasks.
9	Cohen et al. (2017)	Switzerland	To explore the perception of acceptability among community health nurses (CHNs) of an intelligent wireless sensor system (IWSS) for use in daily practice for the detection of health issues in home-dwelling older adults receiving home healthcare	Descriptive and qualitative data from a pilot randomised controlled trial *N* = 17 (CHNs)	Technology acceptance model (TAM)	A majority of the CHNs were dissatisfied with its performance and intrusiveness; they reported multiple difficulties in ease of use of the IWSS technology in daily practice.
10	Özsungur (2022)	Turkey	To analyse the effects of successful aging on technology acceptance and use behaviours via developing a model	Quantitative: survey *N* = 687 (participants in five retirement rest homes) Structural equation model	Unified theory of acceptance and use of technology (UTAUT)	Well-being of older persons is affected by the technology acceptance model in general, except the use of technology
11	Arthanat et al. (2019)	USA	To examine ownership of smart home (SH) technology by older adults and their readiness to adopt SH technology and identify the client factors relating to the adoption	Quantitative: survey *N* = 445 older persons Age = 60+ Stepwise regression model		Marital status, home security and overall Information and Computer Technology (ICT) ownership are predictors of SH ownership, whereas being female, concern over home security and perceived independence contributed to SH readiness. Consideration of the identified client profiles, health and personal factors will strengthen SH integration for ageing in place.
12	Reitsma et al. (2019)	Dutch	To find out the needs that motivate the use of gamified wearables by seniors	Qualitative: interviews: laddering technique *N* = 12 Age: 60–70 and 70+		The need to be healthy and accomplished can be fulfilled by the gamified wearables and motivated seniors to use them. While for some older persons, the safety need for good health is fulfilled by the gamified wearable, other needs are undermined.
13	Lebron et al. (2015)	USA	To observe how the provision of a wireless activity tracker influences the conscious health attitudes and behaviours of older persons	Randomised clinical trial *N* = 6 (older persons) Age: not reported		Older persons anticipated the acceptance of technology due its comfort. However, the perceived benefits of the technology influenced older people’s decision to adopt the technology. Older persons perceived an improvement in their health with the introduction of technology.
14	Portet et al. (2013)	France	To assess the acceptance and objections of smart home voice interface among older persons	Experimental design and interviews *N* = 18 Older persons (*n* = 8); relatives (*n* = 7) and caregivers (*n* = 3) Mean age (older person): 79		Overall acceptance of technologies but with technology anxiety of controlling the lifestyle of older persons.
15	Cajita et al. (2018)	USA	To assess the perceptions of older adults with heart failure regarding the use of mobile technology and identify potential facilitators of and barriers to mHealth adoption	Descriptive exploratory study Semi-structured interviews Content analysis *N* = 5 Age: 66–83 years	Technology acceptance model	Older adults were willing to adopt the mobile health technology, albeit with reservations.
16	Cohen et al. (2016)	Switzerland	To explore the acceptability of intelligent wireless sensor system (IWSS) among home-dwelling older adults in rapidly detecting their health issues	Randomised clinical trial *N* = 34 (older patients)		The IWSS displayed low-to-moderate acceptability among the older participants and their informal caregivers. While older patients were unsatisfied with its ease of use due to multiple obstacles, informal caregivers were more satisfied with its usefulness, having an intention to use IWSS technology.
17	Freiesleben et al. (2021)	Germany	To investigate the barriers to the adoption of locating technologies from a multi-stakeholder professional perspective and explore strategies to optimise adoption	Qualitative: Focus group *N* = 22 Content analysis		Barriers to adoption centred on awareness, technological knowledge, product characteristics and capital investment-based limitations. The study shows that focusing on services to increase digital autonomy and information dissemination strategies has been largely overlooked and may be particularly effective.
18	Peek et al. (2016)	Netherlands	To explore which factors influence the level of use of various types of technology by older adults who are ageing in place and describe these factors in a comprehensive model	Qualitative: Semi-structured interview *N* = 53 Aged: 68–95 years Thematic analysis		Older adults’ perceptions and use of technology are embedded in their personal, social and physical context. Awareness of these psychological and contextual factors is needed to facilitate ageing in place through the use of technology.
19	Turnbull et al. (2021)	Hong Kong	To examine the experiences and perceptions of Hong Kong residents aged over 60 years in relation to mHealth technologies and health literacy	Qualitative: Exploratory design Aged: 60 years Thematic analysis		Older persons were interested in using mHealth technologies. However, their use of digital devices was hindered by a lack of the necessary skills to use these gadgets and their loss of memory.
20	Abdul Rahman et al. (2021)	Malaysia	This study presents a survey that explores older adults' perceptions and expectations toward fall detection devices	A cross-sectional survey *N* = 336 (community-dwelling older adults aged 50 years and older) Chi-square Test		Most older persons expected that features for a fall-detection device include: user friendliness, followed by affordability price and accuracy.
21	Tu and Liu (2021)	China	To examine the moderating effects of subjective well-being (SWB) on the UTAUT model for the elderly’s intention and behaviour regarding the use of gerontechnology	Questionnaire *N* = 487 (older persons) Structural equation model (SEM)	Unified theory of acceptance and use of technology	In all, performance expectancy (PE), effort expectancy (EE) and social influence (SI) positively affected the elderly’s behavioural intention (BI) to use gerontechnology.
22	Ngaruiya et al. (2021)	Kenya	To identify and explore the psychosocial considerations for the gerontechnology design for Kenyan geriatrics	Exploratory case study: Interviews *N* = 8 Age: 65–78 years Thematic analysis		Physical factors related to usability and user experience of older persons when using mobile phones. Psychosocial factors related to the emotional design experienced by older people when using mobile phones.
23	Wilson et al. (2021)	England, Scotland and Wales	To understand older adults’ experiences of using social technology to connect with others	Qualitative exploratory: Semi-interviews *N* = 20 Age: 65 + Thematic analysis		Despite having access to technology for social connection, and using this technology regularly, multiple barriers impacted motivators and skills for use, namely perceived self-efficacy and fear, the culture of online communication, absence of social capital and physical functioning.
24	Jarvis et al. (2020)	South Africa	To investigate communication technology acceptance in older persons living in residential care	A cross-sectional survey *N* = 277 Age: 60 + years Structural equation model (SEM)	Senior technology acceptance model (STAM)	The acceptance of communication technology in this setting was low and predominantly influenced by attitudinal and technological context factors together with age and education.
25	Ha and Park (2020)	South Korea	To investigate the acceptance of technology among older Korean adults with multiple chronic health conditions and examine factors associated with technology acceptance	Quantitative: survey *N* = 226 (community-dwelling older adults) Age: 79.44 years Multiple linear regression model	Senior technology acceptance model (STAM)	Although older Korean adults with multiple chronic conditions displayed good technology acceptance, their age and education level predicted the level of acceptance.

**Table 3:** Quality appraisal for eligible studies

No.	Studies	Quality criteria for quantitative studies				
		Is the sampling strategy relevant to address the research question?	Is the sample representative of the target population?	Are the measurements appropriate?	Is the risk of nonresponse bias low?	Is the statistical analysis appropriate to answer the research question?
1	Chen and Chan (2013)	Yes	Yes	Yes	Yes	Yes
2	Halicka and Surel (2021)	Yes	No	Yes	Can’t tell	Yes
3	Khan et al. (2021)	Yes	Yes	Yes	Yes	Yes
4	Özsungur (2022)	Yes	Yes	Yes	Can’t tell	Yes
5	Arthanat et al (2019)	Yes	Yes	Yes	Yes	Yes
6	Ha and Park (2020)	Yes	Yes	Yes	Can’t tell	Yes
7	Abdul Rahman et al (2021)	Yes	Yes	Yes	Yes	No
8	Tu and Liu (2021)	Yes	Yes	Yes	Yes	Yes
9	Jarvis et al (2020)	Yes	Yes	Yes	Can’t tell	Yes

No.	Studies	Quality criteria for randomised control trials				
		Is randomisation appropriately performed?	Are the groups comparable at baseline?	Are there complete outcome data?	Are outcome assessors blinded to the intervention provided?	Did the participants adhere to the assigned intervention?
10	Lebron et al. (2015)	Yes	Yes	Yes	Can’t tell	Yes
11	Portet et al (2013)	Yes	Can’t tell	Yes	Can’t tell	Yes
12	Cohen et al (2016)	Yes	Yes	Yes	No	Yes

No	Studies	Quality criteria for qualitative studies				
		Is the qualitative approach appropriate to answer the research question?	Are the qualitative data collection methods adequate to address the research question?	Are the findings adequately derived from the data?	Is the interpretation of results sufficiently substantiated by data?	Is there coherence between qualitative data sources, collection, analysis and interpretation?
13	Chen and Chan (2013)	Yes	Yes	Yes	Yes	Yes
14	Chen et al. (2021)	Yes	No	Yes	Yes	Yes
15	Reitsma et al. (2019)	Yes	Yes	Yes	Yes	Yes
16	Cajita et al. (2018)	Yes	Yes	Yes	Yes	Yes
17	Freiesleben et al. (2021)	Yes	No	Yes	No	Yes
18	Ngaruiya et al (2021)	Yes	Yes	Yes	No	No
19	Wilson et al (2021)	Yes	Yes	Yes	Yes	Yes
20	Cohen et al (2017)	Yes	No	Yes	Yes	No
21	Peek et al. (2016)	Yes	Yes	Yes	Yes	Yes
22	Turnbull et al. (2021)	Yes	Yes	Yes	Yes	Yes

No.	Studies	Quality criteria for mixed-method studies				
		Is there an adequate rationale for using a mixed method design to address the research question?	Are the different components of the study effectively integrated to answer the research question?	Are the outputs of the integration of qualitative and quantitative components adequately interpreted?	Are divergences and inconsistencies between quantitative and qualitative results adequately addressed?	Do the different components of the study adhere to the quality criteria of each tradition of the methods involved?
23	Huang et al. (2021)	Yes	Yes	Yes	Yes	Yes
24	Joseph et al. (2018)	Yes	Yes	Yes	No	No
25	Delbreil and Zvobgo (2013)	Yes	No	Yes	No	Yes

### Data analysis

Once the data extraction template was complete, the next stage was to analyse the data. A thematic analysis approach was conducted iteratively by the two independent researchers. The researchers developed themes and presented them to one another to reach a collective understanding. Finally, with an expert researcher’s consultation, the data’s thematic areas were reviewed and concluding themes were formulated.

## Results

First, this section of the review captures the process of collation and the selection of studies. Second, it presents the results on the characteristics of the reviewed studies. Finally, it presents the synthesis of the results thematically according to the research questions.

### Collation and selection of studies

**Fig. 1:**
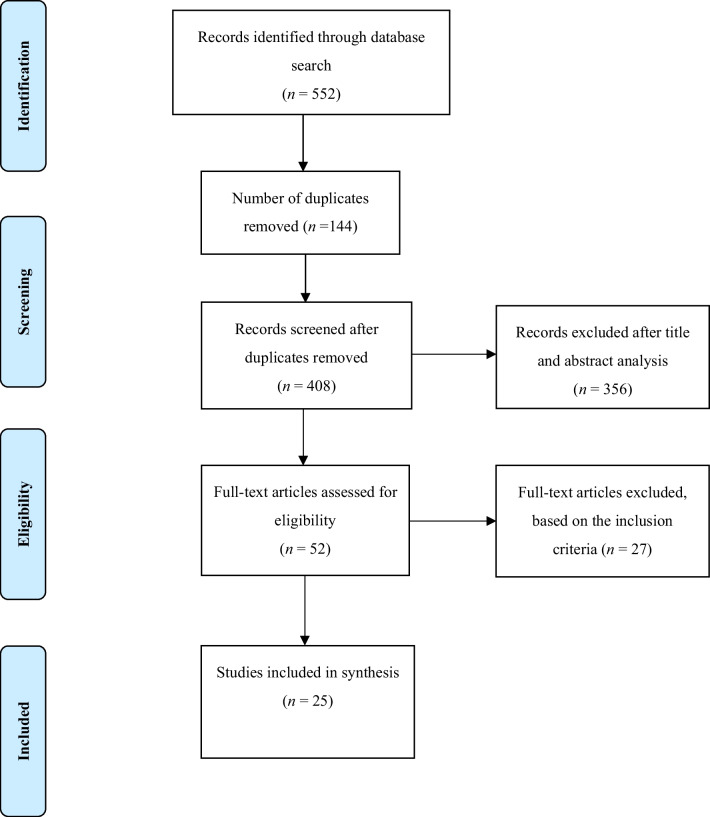
Flowchart showing the search process

The results of the search yielded a total of 552 articles. All articles were exported in CSV Excel file format. After removing duplicate articles, 144 citations remained for title screening. In the next phase, the abstracts of 235 potentially eligible titles were examined. Then, 52 full texts were considered for inclusion, of which only 25 were included in the synthesis and review (Fig. 1).

### Description of reviewed studies

The findings in Table 2 demonstrate that out of the 25 studies, 10 were qualitative, 12 adopted a quantitative approach, and 3 employed a mixed-method design. Pertaining to study context, most studies were conducted in Europe (*n* = 9). This was followed by those conducted in Asia (*n* = 7), Africa (*n* = 5) and North America (*n* = 4). Out of the 25 studies, five focused on caregivers as study participants, and two focused on both older persons and caregivers. The remaining 18 studies extensively focused on older persons.

Moreover, outcomes of gerontechnology adoption on older people’s QoL were conceptualised to encompass healthcare and well-being issues (*n* = 4), as well as health attitudes and behaviours (*n* = 2). Most studies also reported the exact age of older adults to include 55 years or more (*n* = 21). However, one study conceptualised the ages of older workers from 40 years and over. In this article, the justification for including 40-year-old persons was not reported, although it had included a substantial number of older persons aged 50 years and above. In this review, three main theoretical models were espoused: the technology acceptance model (*n* = 5), the senior technology acceptance model (*n* = 1) and unified theory of acceptance and use of technology (*n* = 4). Regarding the study design, most studies were either cross-sectional (*n* = 10) or exploratory (*n* = 8). Only a few studies adopted experimental (*n* = 4) and mixed-method study designs (*n* = 3). A descriptive summary of the reviewed articles is provided in Table 2.

### Gerontechnology adoption by older adults and caregivers

This part of the review discusses the first research question about gerontechnology acceptance by older adults and caregivers. Findings of the thematic analysis revealed three primary themes: evaluation of gerontechnology, proxies of gerontechnology acceptance and barriers to gerontechnology acceptance. The results of each category are provided in the sections that follow.

### Evaluation of gerontechnology

When older adults discussed gerontechnology, studies indicated more positive attitudes (e.g. Lebron et al. 2015; Turnbull et al. 2021; Wilson et al. 2021) than negative attitudes when they perceived the benefits of using gerontechnology. In some studies, gerontechnology received unfavourable attitudes when older adults had no explicit idea of the technology (Abdul Rahman et al. 2021). Beyond attitudes, the studies also emphasised that positive interest was tied to specific gerontechnologies. Gerontechnologies, which improve the overall health of older adults, were preferred when compared to those that were peculiar to some medical conditions of older adults. In this context, providing health information with the aid of digital devices was considered promising and acceptable (Turnbull et al. 2021) compared to other devices, such as fall detection devices (Abdul Rahman et al. 2021), intelligent wireless sensor systems (IWSS) among home-dwelling older (Cohen et al. 2016) or smart home voice (Portet et al. 2013), which may only apply to older adults who experience those peculiar medical conditions.

In contrast, Halicka and Surel (2021) observed that the most important gerontechnologies were those that dealt with older people’s health and safety. Devices related to older adult care and social connectedness took third and fourth place, respectively, followed by mobility, recreational, and health informative devices. Housing and digital accessibility devices were the least important groupings. Some studies demonstrated caregivers’ evaluation of gerontechnology, while others denoted a positive attitude towards gerontechnology to enhance the QoL of older adults (Chen and Chan 2013; Delbreil and Zvobgo 2013; Portet et al. 2013) or reported caregivers’ dissatisfaction with the performance and inappropriateness of the technologies (Cohen et al. 2017).

### Predictors of gerontechnology acceptance

In this section, the following three main sub-themes were identified: technology usability, technology user-friendliness and social factors.

#### Technology usability

One critical motivation for gerontechnology acceptance was technology usability. For instance, studies found that older adults used technology for communication, cooking, supporting daily activities and entertainment (Delbreil and Zvobgo 2013; Huang et al. 2021; Portet et al. 2013; Menghi et al. 2017). Similarly, authors describe gerontechnology acceptance as a caveat to meeting the personal needs of older adults in several domains of life (e.g. Arthanat et al. 2019; Jarvis et al. 2020; Reitsma et al. 2019). These studies are indicative that the need for good health, accomplishment, independence and peace of mind precipitates the usability of technology by older adults.

Other noteworthy studies have highlighted that older adults’ thoughts on technology use were induced by their willingness to invest in technology (Peek et al. 2016) and frequency of use of the technology to increase the frequency of communication with their significant others (Wilson et al. 2021). Besides personal benefits, gerontechnology usability was inextricably tied to social benefits. The literature reports that the perceived usability of gerontechnology would be feasible if it would contribute to the creation of new jobs and bring measurable benefits to the QoL of human health (e.g. Halicka and Surel 2021; Wilson et al. 2021). Apart from older adults, studies report that caregivers’ usefulness of gerontechnology was rated as significant in enhancing the health and safety of older adults (e.g. Cohen et al. 2016; Delbreil and Zvobgo 2013). As reported in the literature, caregivers’ experiences of gerontechnology usability were linked with older adults’ mental health and associated physical disabilities. In sum, the review emphasises that the perceived usability of gerontechnology could have personal and social benefits. In terms of social benefits, older adults stress the contribution of gerontechnology in maintaining their social networks, particularly with their families and children (Peek et al. 2016).

#### Technology user-friendliness

Another key finding connected to the boosters of gerontechnology adoption was user-friendliness. The findings in the review support the argument that perceived ease of use had a significant and positive influence on the usefulness of technology and that ease of use and usefulness predicted positive attitudes towards using gerontechnology (Halicka and Surel 2021). However, in some studies, the user-friendliness of gerontechnology was found not to culminate in the actual usage behaviour of older adults, even when it was expected that usage should be easy and effortless (Chen and Chan 2013).

In contrast to these studies, some studies claim that the user-friendliness of gerontechnology ensures safety and is necessary to guarantee usage behaviour (Delbreil and Zvobgo 2013). Instead, in the literature, some authors were concerned that older adults perceived personal proficiency in operating technology as a determinant of ease of use. For example, depending on the operational proficiency of older adults, entertainment devices, the internet, communication devices and microwave ovens were mentioned in the literature as unique gerontechnology devices that were either user friendly or unfriendly (e.g. Peek et al. 2016). In one pertinent study, perceived ease of use was tied to the ability of technology to connect older adults to significant others (Jarvis et al. 2020).

#### Social factors

In addition to user-friendliness, some studies claim that the availability of social support from significant others is indispensable in technology acceptance and usage (Chen and Chan 2014; Jarvis et al. 2020; Özsungur 2022; Peek et al. 2014). Tu and Liu (2021) added that older adults require proper guidance, assistance and resources from caregivers to use gerontechnology effectively. Beyond the scope of these studies, Chen and Chan (2014) linked older adults’ technology adoption to attaining favourable social outcomes, for instance, the enhancement of one’s image. This implies that using gerontechnology prevents older adults from being labelled outdated.

### Barriers to gerontechnology acceptance

This section discusses six sub-themes that capture the barriers to gerontechnology acceptance. They encompass personal and behavioural factors, economic factors, technological factors, cultural and environmental factors and situational or dispositional factors. Details of the sub-themes are discussed below.

#### Personal and behavioural factors

From the analysis, negative self-evaluated beliefs inhibited gerontechnology acceptance behaviour (Freiesleben et al. 2021; Joseph et al. 2018). For instance, Chen and Chan (2013) found gerontechnology non-usage to be connected to low literacy levels, as it would require older adults to acquire specialised knowledge. The review also established that older adults with lower levels of self-efficacy and anxiety tend to be more likely to use gerontechnology and consider such technology useful and easy to use (Halicka and Surel 2021). Moreover, the authors explain that older adults have greater anxiety and believe they have little control over the technologies. It is evident that older adults feel more anxious and less competent; therefore, they are more resistant to using gerontechnology (Chen and Chan 2014; Jarvis et al. 2020; Wilson et al. 2021). As a result, older adults need assistance when they have difficulties. However, they may also be anxious and reluctant because they do not want to cause inconvenience to their caregivers (Chen and Chan 2013). Older adults have information anxiety caused by their physical health information, considering it to be undermining the tranquillity of their minds (Reitsma et al. 2019). Older adults’ quest to be independent, safe, have personal contact and domestic needs (household chores, hobbies, and voluntary work) were personal indicators in favour of gerontechnology (Huang et al. 2021; Portet et al. 2013).

Beyond older adults’ needs, studies also reiterated that caregivers are anxious that accepting gerontechnology would render older adults indolent (Portet et al. 2013; Reitsma et al. 2019). Arthanat et al. (2019) also identified marital status, home security and internet ownership as the personal predictors of gerontechnology unacceptance. Despite these, the review found that factors such as gender (mostly females), concerns about home security and a sense of independence contributed to gerontechnology adoption. The review also highlighted that cognitive and physical decline was observed to limit older adults’ use of gerontechnology, especially in certain types of technologies, such as household appliances and mobile devices (Ha and Park 2020; Jarvis et al. 2020; Peek et al. 2016). Regarding personal factors, some studies explained that physical features, such as visibility, complexity, feedback, exploration, and recognition, are predominant factors in older adults’ adoption of gerontechnology (Ngaruiya et al. 2021; Wilson et al. 2021). Pertaining to caregivers, the review found that discrepancies between patients’ needs and gerontechnology devices discouraged acceptance (Freiesleben et al. 2021; Huang et al. 2021). It is important to emphasise that socio-demographic factors such as age, health and education predicted gerontechnology acceptance; however, in all, older adults’ adoption intention was moderately low in the review.

#### Economic constraints

Another aspect of the findings is related to the economic constraints on gerontechnology non-usage. Though the role of promotional activities or advertising messages and visuals preceded gerontechnology acceptance (Freiesleben et al. 2021), the review’s findings attributed the non-usage of gerontechnology to cost of the product or service (Chen and Chan 2013, 2014). That is, if the cost of the product or service, training or education fees, and maintenance costs exceed the acceptable range for older adults, users would refuse to use the technology (Peek et al. 2016). In addition, the study discovered that older adults preferred to buy gerontechnology products and services locally because they were cheaper rather than those available on the internet (Peek et al. 2014). Interestingly, the review indicates that older adults receive financial support from family members, implying that the responsibility for elderly care lies with the family rather than the government. Therefore, they may face greater financial constraints when using technology (Chen and Chan 2014).

#### Technological constraints

Gerontechnology properties related to size, language, weight, reliability, and language of others that older adults perceived as unfavourable affected adoption intention (Jarvis et al. 2020; Peek et al. 2016). Detailing technological constraints, Cohen et al. (2017) mentioned that caregivers’ difficulty in managing alert messages from gerontechnology devices increased work demands, particularly for older patients with mental health problems, who were often unable to remember or explain the reason for their behavioural change. This resulted in either under- or overestimation of older patients’ risks of declining health status, resulting in either irrelevant notifications or the absence of notifications in real cases of declining health status.

#### Cultural and environmental factors

Based on the analysis, the review explored environmental and cultural factors constraining gerontechnology acceptance. First, when discussing mobility aids and means of transport, Peek et al. (2016) mentioned that older adults were concerned about road safety, which led to their unacceptance of these types of technology. Second, the literature has found that culture and online communication constrain gerontechnology acceptance (Cohen et al. 2016). Wilson et al. (2021) observed that older adults felt that gerontechnologies, especially those with social benefits, were a useful tool to connect to others but that it did not replicate spending time with one another. Culturally, older people are concerned with their roles in contemporary society and how they can use technology to bridge the intergenerational gap (Ngaruiya et al. 2021). Chen and Chan (2013) found that acceptance and usage of gerontechnology were more difficult for older adults because they were not familiar with their generation compared to the younger generation.

#### Situational or dispositional factors

The thematic analysis also explored situational or dispositional factors in relation to gerontechnology usage. Regarding situational factors, an individual’s current circumstance or situation beyond his or her control was found to impact gerontechnology acceptance (Peek et al. 2016). It has also been found that the use of one type of technology competes with the use of other types (Joseph et al. 2018; Peek et al. 2016). For example, Peek et al. (2016) highlighted that, for some types of technology, older adults’ choice of a landline phone was because they were more familiar with it compared to that of a mobile phone—a technology for the present generation. Abdul Rahman et al. (2021) indicated that the situational barriers included lack of assistance, lack of time, limited exposure to modern technology and inaccessibility and influences of secondary resources. Unlike older adults, caregivers were concerned that most gerontechnology devices could not support caregiving in emergency situations (Freiesleben et al. 2021). In addition to situational constraints, some dispositional factors were also identified from the findings. Chen and Chan (2013) found forgetfulness to use devices as one of the dispositional barriers to gerontechnology. For example, the study mentions that most older adults cannot remember the passwords of their electronic devices.

### Gerontechnology and the QoL of older adults

In this section, the review discusses the thematic findings regarding the impact of gerontechnology adoption on better elderly care and QoL. Two main themes were identified: a healthy lifestyle and social wellness.

#### Healthy lifestyle

Pertaining a healthy lifestyle, the results are ambivalent. The findings were indicative of the fact that gerontechnology adoption affects the QoL of older adults. For example, Delbreil and Zvobgo (2013) assert that caregivers’ confidence in gerontechnology improves older adults’ QoL and lightens the caregivers’ burden. In support of this, Freiesleben et al. (2021) observed that caregivers held favourable views on locating technologies to increase older adults’ QoL. Reitsma et al. (2019) also confirmed that older adults who used gerontechnology to monitor their health had an average active lifestyle, with all of them either walking or cycling regularly. Therefore, the healthy lifestyle of older adults in this instance could cause them to satisfy other life needs when compared with those who are less active. This study further supported the idea that, when provided with information about physical activity, older adults can validate their abilities and qualities and fulfil their need for accomplishment (Reitsma et al. 2019).

Although the impact of quality differs from one type of gerontechnology used to another, a study by Portet et al. (2013) emphasised that the acceptability of a smart home equipped with audio processing technology has enormous potential to ease everyday life for older adults. Moreover, in this study, most of the needs of elderly people were linked to better security at home. Conversely, while this arrangement was expected to produce an independent lifestyle, caregivers were concerned that it would render older people less independent by encouraging an idle lifestyle and further deteriorating their health conditions further (Freiesleben et al. 2021; Portet et al. 2013). Furthermore, findings suggest that using gerontechnology might essentially be a source of risk for older adults who may extensively be exposed to adverse health conditions or a loss of life (Halicka and Surel 2021).

#### Social wellness

It is evident from the findings that gerontechnology was used to enhance existing connections with, as opposed to withdrawal from, society, which has the potential to increase life satisfaction and reduce mental health issues (Wilson et al. 2021). In Wilson et al. (2021), access to and use of gerontechnology, such as digital devices and social media, were valued as tools for social connection. Surprisingly, older adults who were neither lonely nor isolated used technology to connect with others significantly more often than those who experienced loneliness, isolation, or both (Wilson et al. 2021). For older adults who were conservative and preferred face–face communication, Halicka and Surel (2021) mentioned that gerontechnology posed a threat to their social relations to a large extent. However, the review shows that the use of gerontechnology is evident specifically in online visual communication tools as a medium for connecting with friends and family when face-to-face communication is not possible (Wilson et al. 2021).

## Discussion

First, this review has highlighted that older persons’ and caregivers’ attitudes towards gerontechnology are ambivalent. In contrast to many extant studies that conclude on positive attitudes towards gerontechnology usage (e.g. Cohen et al. 2016, 2017; Yow et al. 2018), analysis of the review recognises both positive and negative attitudes towards gerontechnology. Positive attitudes towards gerontechnology are related to the benefits of using gerontechnology, such as abating health, social and family challenges. Unfavourable or negative attitudes towards gerontechnology pertained to older adults’ lack of explicit ideas about the technology, dissatisfaction with its performance and its inappropriateness.

This review has identified that older people’s opinions regarding gerontechnology are important determinants of adoption intentions. This is more crucial, especially when positive attitudes towards gerontechnology may result in higher usage and negative attitudes resulting in lower usage (Chen and Chan 2013, 2014). Thus, efforts to change the negative opinions of older adults regarding gerontechnology should be given greater consideration. While this review underscores the indispensable role of positive attitudes towards gerontechnology adoption, some studies have argued that they may not lead to its usage (Kazanaviˇ and Lesauskait 2019; Lim et al. 2016). Furthermore, substantial number of studies have shown that users’ opinions before and after using gerontechnology are diverse (Merkel and Kucharski 2019; Sundgren et al. 2020). Notably, some studies have demonstrated that the role of social influences on the intention and usage of gerontechnology is substantial in the initial stages of adoption. However, this weakens over time as users familiarise and gain proficiency with the technology (Peek et al. 2016). Similarly, other studies acknowledged that users have positive attitudes towards gerontechnology at the post-usage stage when their health condition improves after usage, although they might have had negative attitudes at the pre-usage stage. The improvement in attitudes towards gerontechnology from pre-usage to post-usage may stimulate continuous usage of the technology (Jansson and Kupiainen 2017; Peek et al. 2016). To facilitate the continued usage and acceptance of gerontechnology products and services, additional longitudinal research is required to better understand users’ full gerontechnology adoption life cycle.

In the review, the self-efficacy and anxiety of older people are significant barriers to adoption of gerontechnology. The results are consistent with extant studies that demonstrate that individuals with lower levels of self-efficacy and higher levels of anxiety towards gerontechnology have a lower acceptance rate of the same (e.g. Latikka et al. 2019; Lee and Tak 2022). However, research indicates that the effects of self-efficacy and anxiety of older people on gerontechnology are more powerful when mediated by user-friendliness and the benefits of the technology (Chen and Chan 2013, 2014; Latikka et al. 2019; Williams and Rhodes, 2016). In contrast to these studies, some studies have argued that older adults’ previous experience and frequency of use of a similar technology tend to increase levels of self-efficacy and reduce levels of anxiety (Kim et al. 2021; Peral-Peral et al. 2020). In general, the review suggests that there are significant differences in self-efficacy and anxiety when discussing technology in general compared to specific types of technology.

As reiterated in the review, financial resources obstruct the adoption of gerontechnology. Mostly, older adults and caregivers find it challenging to purchase and maintain gerontechnology products and services because they are often costly. Consistent with the exchange theory, various studies (e.g. Lee 2014; Lee and Tak 2022) are consistent with the argument that older adults and caregivers are constrained financially as they appraise the costs of technology vis-a-vis its prospective profits in adoption intention and usage. Furthermore, Chen and Chan (2013) found that since the burden for elderly care falls on the families of older people rather than the government, especially those with relatives in care homes, they may face greater financial constraints when it comes to using gerontechnology. However, some studies posit that, unlike caregivers, older adults are often late adopters. When a gerontechnology is introduced, it is labelled as highly innovative, complex, and highly priced; however, as the technology is used over time, it becomes accessible because it tends to be less innovative, simple and cheap (Arthanat et al. 2019; Lee and Kim 2017; Price et al. 2013). Therefore, it is imperative to say that incentives and subsidies provided by policymakers and stakeholders may improve the acceptance rate of gerontechnology.

While gerontechnology usability is critical in adoption intention, the review shows that technological factors pose various hurdles to overcome. Technological factors encapsulated in several design features make gerontechnology easier to control and manage. However, many design systems that have interfaces difficult to read, understand and control fail to comply with usability guidelines (Lee 2014). This is supported by studies conducted in Hong Kong and China, which found that older people do not even know the English alphabet well (Chen and Chan 2013, 2014), making it difficult for them to use electronic equipment with English interfaces. For caregivers, difficulty with technical characteristics resulted in either under- or overestimation of older patients’ risks of declining health status (Frisardi and Imbimbo 2011). The review demonstrates that caregivers do not have complete knowledge of the technical features of gerontechnology, as corroborated in other studies (McHugh and Lawlor 2012; Melkas et al. 2020). Therefore, it is vital to consult caregivers while designing gerontechnology to understand their specific problems and address them comprehensively.

As previously stated, social capital networks are expedient in assisting older adults’ efficient use of gerontechnology. This is substantiated by the fact that older adults with physical and cognitive decline, such as dementia, may be unable to use the gerontechnology (Guisado-Fernández et al. 2019; Kim et al. 2021) without support from social relationships. While the role of social capital in older adults’ care for a health condition cannot be underestimated, it is essential to recognise that gerontechnology adoption and usage is an indication that older adults want to increase autonomy and compensate for age-related health deficiencies (Kohlbacher and Herstatt 2011).

The review demonstrates that gerontechnology acceptance by older adults and caregivers facilitates better elderly care and life quality. For older adults, gerontechnology acceptance and usage depend on the personal and social benefits of products and services. However, gerontechnology usage for caregivers is found to be contingent on the efficiency of the product or service, which can enhance the health and safety of older adults. The implication is that older adults and caregivers are more likely to adopt gerontechnology when they expect it to result in favourable outcomes. This raises the possibility that more optimistic users about any specified gerontechnology perceive it as more beneficial and easier to use compared to less favourable users (Godoe and Johansen 2012).

Moreover, these findings also support the notion that gerontechnology offers older adults, particularly cohorts of older persons in care homes, the opportunity to sustain their independence by ageing actively and ageing in place (Ollevier et al. 2020). This necessitates that social caregivers understand these new technology and the potential benefits for older adults’ health promotion and assistance (Schmitter-Edgecombe et al. 2013). Thus, the studies examined in this review highlight the importance of professional training and development for social caregivers regarding the use of existing and emerging gerontechnologies to create more ecologically valid, impartial, and frequent measures of change when monitoring older people’s healthy functioning.

### Implications for further research agenda

Overall, both older workers and caregivers agree that a positive attitude towards gerontechnology is a means to enhance the QoL of older people. However, studies on the attitudes and perceptions of social caregivers and health professionals towards gerontechnology acceptance have received little attention so far. Therefore, future studies should investigate the factors of gerontechnology acceptance or unacceptance by social caregivers and health professionals. The paper highlights that the most important gerontechnology products and services are those that deal with older people’s health and safety.

Further research can also investigate the structural factors that differ between these technologies and their stages of usage among older adults and social caregivers. More specifically, a longitudinal study is required on how changes in the factors identified in this review affect older adults’ and social caregivers’ attitudes and beliefs regarding the use of gerontechnology.

It is invariably reasonable to appreciate that various studies identified in the review proposed theoretical models to explain older people’s adoption of gerontechnology. Factors identified in frameworks such as the technology adoption model (TAM), senior technology adoption model (STAM) and unified theory of acceptance and use of technology (UTAUT) were found to impact gerontechnology adoption by both older adults and caregivers. However, these models acknowledge that some factors may not be able to predict gerontechnology acceptance and usage. For instance, consistent with TAM, STAM and UTAUT, some studies in the review underscored the influence of attitudinal factors such as technology usability and user-friendliness in adoption. However, other studies found that personal, technological, and environmental factors were imperative in adoption and usage rather than attitudinal factors. This suggests the relevance of other factors that explain the unacceptance or acceptance of gerontechnology, regardless of the adoption intention of older people and social caregivers.

Thus, the review identified many mediating factors that explain the relationship between gerontechnology acceptance and the QoL of older adults. Hence, further studies employing quantitative methodology can investigate the moderating or mediating relationships between these factors and the strength of their relationships with each other. For example, a positive self-perception of ageing and satisfaction with life would increase the possibility of using technology. In return, using technology can also increase older users’ well-being and self-evaluation. Future qualitative studies can deepen studies on older adults’ and social caregivers’ reasons for using or not using gerontechnology, regardless of their adoption intentions. In addition, more qualitative research is needed to better understand how older adults evaluate and decide between the various gerontechnology options available to them when faced with challenges in the domain of independent living. The paper also supports that it is important to study the use of technology by older adults’, including understudied populations, such as the oldest-old and rural older adults, since these populations may have different health and technology needs.

### Strengths and limitations

Our study has several strengths. First, this review offers a comprehensive evidence on gerontechnology acceptance and usage by focusing on the perspectives of both older persons and social caregivers, compared to many systematic reviews, which are predominantly themed on the older population. Second, the inclusion of studies that evaluate the effect of gerontechnology usage by older individuals and social caregivers contributes to scholarship by presenting systematic evidence that goes beyond gerontechnology usage intentions or adoption. In addition to theoretical evidence on gerontechnology intention or behaviour, evidence from this review informs policy or practice to ensure better elderly care and quality of life. Furthermore, the inclusion of both quantitative and qualitative study designs improves the quality of this review by removing any potential methodological bias and extending the scope and depth of evidence on the topic.

However, we acknowledge that our review has some limitations. First, we acknowledge that since this study was limited to publications in English language, there remains a possibility of missing other relevant studies and insights in some languages. In addition, the search strategy was exclusively restricted to peer-reviewed publications, excluding possibly relevant dissertations, conference presentations and book chapters. As we adopted a thematic approach in analysing the studies, we admit that there were no statistical or other quantitative techniques of analyses. Nonetheless, the thematic analysis goes beyond the narrative approach of mere descriptions and summary of the main features of included studies. Rather, the review explored the similarities and differences between studies, assessed their contributions to extant literature, and the practice or policy implications for future discourse on gerontechnology.

## Conclusion

Through a systematic approach, this paper contributes to scholarship by extending knowledge of the experiences of both older adults and social caregivers regarding the acceptance and unacceptance of gerontechnology. This paper concludes that the impact of gerontechnology acceptance on both older adults and social caregivers is highly dependent on certain personal, socio-cultural, technological and physical factors. Furthermore, since older adults and social caregivers constitute two heterogeneous groups, a unitary or all-purpose policy approach for gerontechnology and a better QoL may be ineffective.
